# Effect of Gum Tragacanth-Sodium Alginate Active Coatings Incorporated With Epigallocatechin Gallate and Lysozyme on the Quality of Large Yellow Croaker at Superchilling Condition

**DOI:** 10.3389/fnut.2021.812741

**Published:** 2022-01-18

**Authors:** Juxin Pei, Jun Mei, Huijie Yu, Weiqiang Qiu, Jing Xie

**Affiliations:** ^1^College of Food Science and Technology, Shanghai Ocean University, Shanghai, China; ^2^National Experimental Teaching Demonstration Center for Food Science and Engineering, Shanghai Ocean University, Shanghai, China; ^3^Shanghai Engineering Research Center of Aquatic Product Processing and Preservation, Shanghai, China; ^4^Shanghai Professional Technology Service Platform on Cold Chain Equipment Performance and Energy Saving Evaluation, Shanghai, China

**Keywords:** epigallocatechin gallate, lysozyme, active coating, large yellow croaker, shelf life

## Abstract

This research was done to investigate the synergistic interactions of the gum tragacanth (GT)–sodium alginate (SA) active coatings, incorporated with epigallocatechin gallate and lysozyme, on the quality of large yellow croaker *(Larimichthys crocea*) during superchilling storage at −3°C. Results showed that the GT-SA active coatings, containing epigallocatechin gallate [EGCG (E), 0.32% w/v], and lysozyme [LYS (L), 0.32% w/v] have reduced the total viable count, psychrophilic bacteria, and *Pseudomonas* spp. by about 1.55 log CFU/g, 0.49 log CFU/g, and 1.64 log CFU/g compared to the control at day 35. The GT-SA active coatings containing EGCG and LYS were effective in lowering the formations of off-odor compounds such as total volatile basic nitrogen (TVB-N), malondialdehyde (MDA), and off-favor amino acid (histidine). The solid phase microextraction gas chromatography-mass spectrometer (SPME-GC/MS) was applied to characterize and to quantify the volatile compounds of large yellow croaker samples during superchilling storage, while the relative content of the fishy flavor compounds (including 1-octen-3-ol and acetoin) was significantly reduced in the active coatings treated samples. Furthermore, the GT-SA active coatings containing EGCG and LYS treatments was found to be more effective in retarding the migration of water based on magnetic resonance imaging (MRI) results and in maintaining the organoleptic quality of large yellow croaker in superchilling storage at −3°C according to the sensory evaluation results. The results showed that the GT-SA active coating containing EGCG and LYS was effective to be used as a fish preservative to improve the quality and to prolong the shelf life of large yellow croaker in a superchilling storage for at least 7 days.

## Introduction

Large yellow croaker (*Larimichthys crocea*) is a marine fish with a delicate flesh and a delicious flavor. It is deemed to be one of the most valuable, economical, and commercial fishes in China (1). However, the high nutrition and water content of fresh large yellow croaker flesh makes it spoil easily by microorganisms (2), and its high fat content is prone to oxidation, resulting in undesirable flavor (3). Therefore, it is necessary to take some measures to inhibit or to delay the microbial growth and lipid oxidation to slow down the reduction of the fish quality and to prolong the shelf life of large yellow croakers. Low-temperature preservation is a common way to preserve fish and fish products, however, low-temperature does not adequately inhibit the growth of microorganisms and the occurrence of terrible chemical reactions. The synergistic interactions of different non-chemical preservatives on the preservation of fresh fish have attracted more and more attention. In addition, for the past few years, some researchers have focused on the addition of different non-chemical preservatives, including active coatings, to prolong the preservation life of fish (4).

Sodium alginate (SA) is a natural, linear, and hydrophilic polysaccharide consisting of β-1,4 glycosidic bond linked α-L-guluronic glucuronic acid, and β-D-mannuronic acid (5). It has good film-forming attributes and can be used to make SA-based active coatings. However, SA shows some disadvantages, such as poor thermal stability (6) and low encapsulation efficiency (7) in hydrogel a matrix. Some studies have found that blending SA with other biopolymers can mitigate the adverse effects of SA (6, 8, 9). Gum tragacanth (GT) is a natural, highly branched, and hydrophilic polysaccharide which is produced from the branches and stems of Asiatic *Astragalus* species (10). As a safe biopolymer, GT has unique attributes, such as non-toxicity, biocompatibility, and thermal stability (11). Apoorva et al. (12) reported that the GT-SA composite biopolymers could be used as a packaging for phenolic substances, due to its ability to increase encapsulation efficiency of phenolic substances in a simulated intestinal fluid.

Epigallocatechin gallate [EGCG (E)], the main polyphenol component of green tea (13), has been researched to possess antimicrobial and antioxidant properties. It has been demonstrated that EGCG *in vitro* has significant antibacterial activity against a variety of Gram-positive and Gram-negative bacteria. The EGCG bonds and attacks the lipid regions of Gram-positive and Gram-negative cytoplasmic membranes, contributing to the growth inhibition and death of microorganisms (14, 15). Furthermore, EGCG could keep foods from oxidative deterioration through different mechanisms, including inhibition of hydrogen atom transfer, electron transfer, and the chelation of catalytic metals (16). As a promising natural plant-derived polyphenol preservative, EGCG is safer and less harmful than synthetic chemical preservatives (17). Cao et al. (18) reported that the gelatin coatings containing EGCG have lowered the count and the relative abundance of microorganisms and have also retarded the rate of lipid oxidation in tilapia during refrigeration storage. Some researchers also showed that the EGCG could synergize with antibiotics such as penicillin in terms of bacteriostatic effects (13), hence, a possible way to combine EGCG with other natural compounds to achieve a greener fresh-keeping effect.

Lysozyme [LYS (L)], a hydrolase widely present in egg whites, has attracted extensive attention within the food industry since the twenty-first century (19). The LYS has a great potential for food preservation owing to its stability over a wide pH and a temperature range (20). The β-1,4 glycosidic bond between N-acetylglucosamine and N-acetylmuramic acid in the bacterial cell wall can be hydrolyzed by LYS. Therefore, LYS shows strong antibacterial activity against Gram-positive bacteria, but this ability is weaker in Gram-negative bacteria due to the protection of the cell wall by an outer membrane of the Gram-negative bacteria (21, 22). Given that, LYS has a limited application in the aquatic products industry, but this restriction may be eliminated by combining with other preservatives that can increase its antibacterial spectrum. Notably, the combination of LYS and succinic acids could be used as a food preservative to efficiently inhibit the growth of *L. monocytogenes* (23); the combination of resveratrol, LYS, and chitosan could effectively prolong the shelf life of *Sciaenops ocellatus* by 8 days (24); and the combination of tea polyphenols, LYS, and chitosan could significantly extend the preservation life of pomfret filet (25). According to the above-mentioned views, one of the most powerful methods to extend the shelf life of fish filets is the use of active coatings containing EGCG and LYS in a low-temperature storage.

Therefore, the objective of the present study was to investigate the effects of the preservative of GT and SA-based active coatings containing EGCG and LYS on large yellow croaker during the superchilling storage at −3°C in terms of microbiological analysis, water distribution and migration, total volatile basic nitrogen (TVB-N), free amino acids (FAAs), and volatile compounds (VOCs) analysis.

## Materials and Methods

### Materials and Chemicals

The compound preservatives were composed of epigallocatechin gallate (EGCG; purity ≥98%; Yuanye, Shanghai, China) lysozyme (LYS; ~70000 U/mg; Sigma–Aldrich, Shanghai, China). Gum tragacanth (GT) and sodium alginate (SA) were obtained from Aladdin, Shanghai, China. Glycerol, as a plasticizer for improving the flexibility of active coatings, was obtained from Macklin Biochemical Co, Ltd (Shanghai, China).

### Preparation of Active Coatings Containing EGCG and LYS

Gum tragacanth-sodium alginate (GT-SA) active coatings containing EGCG and/or LYS solutions were prepared according to the method of Liu et al. (26). The EGCG (0.16 and 0.32%, w/v, respectively), and LYS (0.04 and 0.32%, w/v, respectively), were dissolved in a deionized water to get 100 ml of EGCG and LYS solutions [the concentrations of EGCG and LYS were identified by our preliminary experiments (data not shown)]. Gum tragacanth (GT) (0.5% w/v), SA (1.5% w/v), and glycerol (0.6% w/v) were dissolved in a deionized water at 45°C and were stirred continuously for 4 h to get 900 ml of mixed solutions. Then, the mixture was dissolved in the prepared EGCG and/or LYS solutions (total 1 L) and was stirred for 30 min. Finally, the coating solutions were ultrasonically treated (XEB-1000-P, Xiecheng, Shandong, China) for 10 min at 800 W to homogenize and to degas. The final active coating solutions were signed as GT-SA, 0.04L, 0.16E, 0.16E + 0.04L, 0.16E + 0.32L, 0.32E + 0.04L, and 0.32E + 0.32L, respectively.

### Preparation of Large Yellow Croakers and Samples Treatment

A total of 83 live large yellow croakers were obtained from Xiaoma Fresh Market (Shanghai, China) with an average weight of 550 ± 20 g. The fishes were treated with ice for 20 min to stun them, and then were killed before the guts and gills were discarded. After that, they were fileted with an average weight of 110 ± 10 g and were washed with a sterile normal saline (0.85% w/v) completely. Three random large yellow croaker samples were selected on day 0 (initial sampling point) for the determination of the basic quality indexes (27). The remaining large yellow croaker samples were divided evenly into 8 batches as listed in Table 1. Each batch of large yellow croaker samples were submerged in the corresponding freshly prepared coating solutions (the ratio of samples to solution, 1:3, w/v) for 15 min and then dried for 1 h at 4°C with 50% relative humidity. After that, all samples were packaged in a sterile bag (not under vacuum) and stored at −3 ± 0.2°C in a thermostatic chamber (BPS-250CB, Yiheng, Shanghai, China). Large yellow croaker samples were selected randomly for analysis on 0, 7th, 14th, 21st, 28th, and 35th days.

**Table 1:** Sample preparation with different epigallocatechin gallate (EGCG) and/or lysozyme (LYS) contents.

**Sample**	**Treatment**		
	**EGCG content (%, w/v)**	**LYS content (%, w/v)**	**GT-SA**
CK	–	–	–
GT-SA	–	–	+
0.04L	–	0.04	+
0.16E	0.16	–	+
0.16E + 0.04L	0.16	0.04	+
0.16E + 0.32L	0.16	0.32	+
0.32E + 0.04L	0.32	0.04	+
0.32E + 0.32L	0.32	0.32	+

*–, not included; +, included*.

### Microbiological Analysis

Five grams of large yellow croaker samples were mixed thoroughly with 45 ml normal saline (0.85% w/v) and then subjected to serial dilutions. The total viable counts (TVC) of *Pseudomonas* spp. were determined by incubation on the medium of plate count agar and *Pseudomonas* CFC selective agar for 2 days at 30°C. The psychrophiles bacteria were determined by incubation on the medium of plate count agar for 7 days at 4°C (27).

### Water Distribution and Migration

The proton relaxation experiments were carried out by the low-field nuclear magnetic resonance (LF-NMR) technology according to Li et al. (28). The dorsal muscles of the large yellow croaker samples were cut into cubes (2 cm × 2 cm × 1.5 cm pieces, about 5 g), packaged in polyethylene cling film, and then measured in triplicate by LF-NMR analyzer (MesoMR23-60H.I, Niumag, Suzhou, China). To get the pseudo-color pictures of the proton density weight of large yellow croaker, nuclear magnetic resonance imaging (MRI) experiments were also conducted. The measured parameters were as follows: (a) Proton resonance frequency: 21 MHz; (b) Slice width: 3.5 mm; (c) Time echo: 0.5 ms; and (d) Time repetition: 2,000.

### Water-Holding Capacity Analysis

Water-holding capacity (WHC) values were determined based on the method of Merlo et al. (29). Briefly, approximately 3 g of large yellow croaker samples was taken from the dorsal portion, weighed (W_0_), and centrifuged at 2,653 g for 10 min at 4°C. The samples were weighed again (W_1_) after centrifugation and their ability to hold water was expressed as WHC, which was calculated as follows:


$$\begin{array}{l} WHC\;\left(\%\right)=\frac{W_{1}}{W_{0}}\times 100\% \end{array}$$

### Cooking Loss Analysis

The measurement of cooking losses refers to the method of Sun et al. (30). In brief, ~2 g of large yellow croaker samples from the dorsal portion was weighed (C_0_) and packed in separate polyethylene bags. The samples were boiled for 15 min at 85°C and then remove the water on the surface before the samples were weighed again (C_1_). Cooking loss was calculated according to the following equation:


$$\begin{array}{l} Cooking\;lose\;\left(\%\right)=\frac{C_{0}-C_{1}}{C_{0}}\times 100\% \end{array}$$

### pH Analysis

With slight modifications, the pH values were measured according to the method of Kim et al. (31). Two grams of minced large yellow croaker samples were mixed thoroughly with 20 ml of ultrapure water and then centrifuged at a speed of 2,653 g for 1 min at 4°C. The supernatant after centrifugation was used to determine the pH values with a pH meter (PB-10, Sartorius, Goettingen, Germany).

### Total Volatile Basic Nitrogen (TVB-N) Analysis

Total volatile basic nitrogen (TVB-N) values were measured by referring to the method recommended by Mei et al. (32). Five grams of minced large yellow croaker samples were accurately weighed into a Kjeldahl nitrogen flask and mixed with 1.5 g of MgO. The mixture was then measured using Kjeldahl nitrogen tester (Kjeltec 8400, Foss, Hillerød, Denmark).

### Extraction of Salt Soluble Protein Solution and Determination of Salt Soluble Protein Content

The extraction of salt soluble protein solution was performed based on the method of Lv et al. (33). Exactly 2 g of minced large yellow croaker samples was fully mixed with 20 ml of cold Tris-buffer A (0.05 M KCl, pH 7). The mixture was centrifuged at 10,614 g for 15 min at 4°C, and then the supernatant was removed. This step was repeated once again before the collected precipitate was fully mixed again with 20 ml of cold Tris-buffer B (0.6 M NaCl, pH 7) and allowed to stand for 3 h at 4°C. After that, the final mixture was centrifuged at 10,614 g for 15 min at 4°C, and the remaining liquid after removal of the precipitate was a salt soluble protein solution. The supernatant was then centrifuged at 10,614 g for 15 min at 4°C. The content of salt-soluble protein was taken by the assay of Biuret protein method (34).

### Evaluation of Malondialdehyde (MDA) Content

The content of malondialdehyde (MDA) was measured by the method of Ohkawa et al. (35). Briefly, 2 g of minced large yellow croaker samples from the belly was accurately taken and homogenized by adding 18 ml of normal saline (0.85% w/v). The homogenate was mixed with an MDA analysis solution [composed of thiobarbituric acid, acetate buffer (pH 3.5), and sodium dodecyl sulfate] and then reacted at 100°C for 60 min at 532 nm to measure the absorbance. The MDA content was expressed as nmol/mg prot of large yellow croaker samples.

### Free Amino Acids Analysis

The method of FAAs was described by Liu et al. (26). Two grams of minced large yellow croaker samples was accurately weighed and mixed thoroughly with 10 ml of 5% trichloroacetic acid. The mixture was centrifuged at 10,000 g for 15 min at 4°C, and the precipitate was removed. Centrifugation and removal of precipitate was repeated once again before the combined supernatant, after two centrifugations, was diluted to 25 ml and filtered through a 0.22 μm membrane. The final filtrate was used to determine FAAs in the amino acid analyzer (L-8800, Hitachi, Tokyo, Japan).

### Taste Activity Value Analysis

Taste activity value (TAV) was calculated referring to the method of Qi et al. (36). TAV is the ratio of taste compounds' concentration and its threshold value. Amino acids with TAV > 1 are understood to be the main contributors to taste.

### Headspace SPME Gas Chromatography-Mass Spectrometer Analysis

The method of Liu et al. (26) was used to characterize and quantify the volatile chemicals (VOCs) of large yellow croaker samples by SPME-GC/MS, with slight modifications. Five grams of minced large yellow croaker samples was precisely taken and mixed with 5 ml of saturated NaCl solution in a headspace flask. The VOCs were determined by comparison with the mass spectra included in NIST 2011. The specific parameters are set as follows: (a) Instrument: GCMS TQ8040 (Shimadzu, Inc., Japan); (b) Column: Rtx-wax (30 m × 0.25 mm, 0.25 μm); (c) Solid phase extraction conditions: Blue extraction head with Phase Thickness 120 um, phase length 20 mm, Carbon WR/PDMS; Equilibrium time 15 min, extraction temperature 50 °C, extraction time 30 min and desorption time 5 min; (d) GC conditions: Injector temperature at 260°C, do not tap; Carrier gas: Helium (99.999%); Column temperature: 40°C for 5 min and increase to 220°C at 5°C/min, do not hold, then rise to 250°C at 20°C/min and hold for 2.5 min; Flow rate: 1 ml/min; (e) Mass spectrum conditions: Ion source temperature at 230°C; Ionization mode: EI, 70eV; Quality range: 20–400.

### Sensory Evaluation

The sensory evaluation of large yellow croaker samples at different time points was carried out referring to the method of Luan et al. (37). A trained 10-member panel (six female and four males between 22 and 45 years old) assessed the samples. Samples with different treatments were randomly taken out of the refrigerator and thawed, and then immediately presented to the panelists (simultaneous evaluation by each panelist). The sensory evaluation was done on each test day and was based on a ten-point scale to determine morphology (10, intact; 1, very loose), color discoloration (10, bright; 1, extremely dull), odor (10, extremely desirable; 1, extremely fishy), and elasticity (10, quick rebound after finger pressure; 1, depression after finger pressure) of the large yellow croaker samples. The panelists were also presented with a freshly thawed fish sample that had been stored at −40°C throughout the experiment which served as the control sample. The sensory scores of each filet qualities (weight values: 0.2, 0.3, 0.3, and 0.2, respectively), were added together to provide an overall sensory score (total score: 10). When the overall sensory score fell below 6, the shelf-life criterion expected that rejection would occur.

### Statistical Analysis

All experiments were performed in triplicates unless otherwise specified. The SPSS 24.0 was used to conduct a one-way ANOVA on the data, and the final results were expressed as means ± SD. Data were subjected to Duncan's *post-hoc* test. Differences were considered statistically significant at *P* < 0.05. Graphs were produced using Origin 2021.

## Results and Discussion

### Microbiological Analysis

The growth counts of TVC, psychrophiles bacteria, and *Pseudomonas* spp. of large yellow croaker samples during superchilling storage are listed in Table 2. The initial TVC count was 4.9 log CFU/g, reflecting that the starting quality of the samples was good based on an acceptable maximum count of 5 log CFU/g for fresh fish (38). The trends in TVC counts were similar for the different treatments. Firstly, the TVC counts declined in fish muscle from 0 day to 7th day during superchilling storage. This observation can be illustrated by the fact that the superchilling temperature is at the maximum ice crystal generation zone. The water in the microbial fluid freezes and expands in volume, while the microorganism is also squeezed externally due to the increased volume of water-generated ice, causing bacterial rupture and death. Then, the TVC counts continued to increase during the subsequent superchilling storage. On the 28th day, CK and SA reached the “shelf-life” limit of 7.0 log CFU/g (39). On the 35th day, the CK (control check), GT-SA, 0.04L, and 0.16E samples all exceeded the limit of the TVC counts and had to be removed. For the 0.16E + 0.04L, 0.16E + 0.32L, 0.32E + 0.04L, and 0.32E + 0.32L samples, the TVC counts did not reach the limit throughout the superchilling storage and were visibly lower than the other samples at any of sampling points (Table 2). The application of combined EGCG and LYS could delay the total microbial growth in large yellow croaker superchilling storage due to the remarkable antibacterial effect. In a related study, Li et al. (24) also demonstrated that the combination of resveratrol and LYS had a good antimicrobial activity to inhibit the TVC growth. Similar tendencies were also observed in this research on the increase of psychrophiles bacteria and *Pseudomonas* spp. Psychrophiles bacteria could produce the metabolic compounds, such as sulfur-containing volatile compounds, ketones, and biogenic amines, resulting in deterioration of the odor, flavor, and texture of the large yellow croaker samples (40). The growth curve of psychrophiles bacteria in each group (Table 2) was similar to the corresponding TVC counts, suggesting that EGCG-LYS treatments could effectively inhibit the growth of psychrophiles bacteria. Often, the *Pseudomonas* spp. is considered as a specific spoilage bacterium of aquatic products. For example, Bono et al. (41) found that the specific spoilage bacteria of both European anchovy and sardine were *Pseudomonas* spp. In this research, the total *Pseudomonas* spp. count for CK, GT-SA, 0.04L, 0.16E, 0.16E + 0.04L, 0.16E + 0.32L, 0.32E + 0.04L, and 0.32E + 0.32L samples were 6.72, 6.76, 6.61, 6.56, 5.45, 5.23, 5.28, and 5.08 log CFU/g on the 35th day (Table 2), respectively. It was also found that the application of LYS alone had low antibacterial effect on *Pseudomonas* spp. due to its poor inhibitory effect on Gram-negative bacteria (21, 22). However, the count of *Pseudomonas* spp. was significantly reduced when the compound preservatives were used. The hydrogen peroxide produced by EGCG was found to be the main cause of damage to the cell wall of Gram-negative bacteria by Cui et al. (42). In addition, the reason for the good synergistic inhibitory effects of EGCG-LYS on *Pseudomonas* spp. (Gram-negative bacteria) may have been caused by an extremely high antibacterial property when hydrogen peroxide and LYS were present at the same time, which was confirmed in dairy products (43). Huang et al. (44) also reported that the nano-sanitizers, in which hydrogen peroxide and LYS were the major active components, showed significant inhibition of *E. coli* and *Pseudomonas* spp. populations on spinach. Active coatings containing EGCG and LYS could inhibit a more successful growth of bacterial, thus, extending the shelf life for at least 7 days of large yellow croaker during superchilling (45).

**Table 2:** Changes in microbial load of large yellow croaker samples during superchilling storage.

**Organism**	**Treatment**	**Changes in microbial load of large yellow croaker (log CFU/g)**					
		**0 d**	**7 d**	**14 d**	**21 d**	**28 d**	**35 d**
Total viable counts	CK	4.90 ± 0.08^a^	4.21 ± 0.16^c^	5.48 ± 0.10^a^	6.40 ± 0.21^a^	7.01 ± 0.20^a^	7.79 ± 0.21^a^
	GT-SA	4.90 ± 0.08^a^	4.89 ± 0.22^a^	5.57 ± 0.20^a^	6.78 ± 0.09^b^	7.10 ± 0.21^a^	7.83 ± 0.27^a^
	0.04L	4.90 ± 0.08^a^	4.51 ± 0.21^bc^	5.05 ± 0.08^b^	5.99 ± 0.11^c^	6.60 ± 0.15^b^	7.31 ± 0.13^b^
	0.16E	4.90 ± 0.08^a^	4.83 ± 0.27^ab^	5.09 ± 0.09^b^	6.01 ± 0.12^c^	6.62 ± 0.14^b^	7.48 ± 0.15^ab^
	0.16E + 0.04L	4.90 ± 0.08^a^	3.79 ± 0.23^d^	4.90 ± 0.11^b^	5.70 ± 0.10^c^	6.33 ± 0.16^bc^	6.79 ± 0.10^c^
	0.16E + 0.32L	4.90 ± 0.08^a^	3.63 ± 0.10^d^	4.85 ± 0.24^b^	5.27 ± 0.26^d^	6.03 ± 0.19^d^	6.54 ± 0.25^cd^
	0.32E + 0.04L	4.90 ± 0.08^a^	3.68 ± 0.08^d^	4.88 ± 0.20^b^	5.38 ± 0.20^d^	6.06 ± 0.21^cd^	6.66 ± 0.22^c^
	0.32E + 0.32L	4.90 ± 0.08^a^	3.50 ± 0.18^d^	4.25 ± 0.19^c^	5.22 ± 0.24^d^	5.82 ± 0.19^e^	6.24 ± 0.20^d^
Psychrophiles bacteria	CK	4.53 ± 0.11^a^	4.48 ± 0.10^a^	4.60 ± 0.11^ab^	4.97 ± 0.13^a^	5.40 ± 0.05^ab^	5.75 ± 0.20^a^
	GT-SA	4.53 ± 0.11^a^	4.48 ± 0.11^a^	4.66 ± 0.17^a^	5.00 ± 0.17^a^	5.48 ± 0.13^a^	5.79 ± 0.14^a^
	0.04L	4.53 ± 0.11^a^	4.25 ± 0.11^bc^	4.48 ± 0.11^abc^	4.83 ± 0.17^ab^	5.38 ± 0.13^ab^	5.66 ± 0.03^a^
	0.16E	4.53 ± 0.11^a^	4.34 ± 0.15^ab^	4.53 ± 0.16^abc^	4.86 ± 0.11^ab^	5.39 ± 0.18^ab^	5.69 ± 0.09^a^
	0.16E + 0.04L	4.53 ± 0.11^a^	4.23 ± 0.15^bc^	4.38 ± 0.17^bcd^	4.70 ± 0.10^b^	5.18 ± 0.08^bc^	5.40 ± 0.10^b^
	0.16E + 0.32L	4.53 ± 0.11^a^	4.15 ± 0.14^bc^	4.33 ± 0.10^cd^	4.61 ± 0.15^b^	5.09 ± 0.11^c^	5.36 ± 0.08^b^
	0.32E + 0.04L	4.53 ± 0.11^a^	4.21 ± 0.06^bc^	4.35 ± 0.12^cd^	4.68 ± 0.09^b^	5.16 ± 0.20^bc^	5.39 ± 0.06^b^
	0.32E + 0.32L	4.53 ± 0.11^a^	4.10 ± 0.08^c^	4.21 ± 0.06^d^	4.31 ± 0.16^c^	4.85 ± 0.12^d^	5.26 ± 0.09^b^
*Pseudomonas* spp.	CK	3.74 ± 0.14^a^	3.44 ± 0.17^ab^	4.86 ± 0.20^ab^	5.75 ± 0.17^a^	6.12 ± 0.11^a^	6.72 ± 0.16^a^
	GT-SA	3.74 ± 0.14^a^	3.64 ± 0.11^a^	4.97 ± 0.18^a^	5.79 ± 0.25^a^	6.26 ± 0.08^a^	6.76 ± 0.15^a^
	0.04L	3.74 ± 0.14^a^	3.54 ± 0.21^a^	4.76 ± 0.17^abc^	5.45 ± 0.16^b^	6.08 ± 0.14^ab^	6.61 ± 0.10^a^
	0.16E	3.74 ± 0.14^a^	3.46 ± 0.27^ab^	4.58 ± 0.24^bcd^	5.40 ± 0.14^bc^	5.78 ± 0.15^bc^	6.56 ± 0.18^a^
	0.16E + 0.04L	3.74 ± 0.14^a^	3.23 ± 0.26^b^	4.40 ± 0.22^cde^	5.21 ± 0.18^bcd^	5.54 ± 0.15^cd^	5.45 ± 0.12^b^
	0.16E + 0.32L	3.74 ± 0.14^a^	3.15 ± 0.18^b^	4.15 ± 0.19^e^	4.0.99 ± 0.19^de^	5.08 ± 0.04^e^	5.23 ± 0.11^bc^
	0.32E + 0.04L	3.74 ± 0.14^a^	3.18 ± 0.19^b^	4.30 ± 0.25^de^	5.10 ± 0.22^cde^	5.27 ± 0.10^de^	5.28 ± 0.15^bc^
	0.32E + 0.32L	3.74 ± 0.14^a^	3.10 ± 0.24^b^	4.13 ± 0.19^e^	4.81 ± 0.15^e^	4.98 ± 0.21^de^	5.08 ± 0.20^c^

*CK, uncoated; GT-SA, GT-SA coating without preservatives; 0.04L, GT-SA coating with 0.04 mg·mL^−1^ LYS; 0.16E, GT-SA coating with 0.16 mg·mL^−1^ EGCG; 0.16E + 0.04L, GT-SA coating with 0.16 mg·mL^−1^ EGCG and 0.04 mg·mL^−1^ LYS; 0.16E + 0.32L, GT-SA coating with 0.16 mg·mL^−1^ EGCG and 0.32 mg·mL^−1^ LYS; 0.32E + 0.04L, GT-SA coating with 0.32 mg·mL^−1^ EGCG and 0.04 mg·mL^−1^ LYS; and 0.32E + 0.32L, GT-SA coating with 0.32 mg·mL^−1^ EGCG and 0.32 mg·mL^−1^ LYS*.

*Same superscript letters for mean values within a column indicate no significant differences (P > 0.05)*.

### LF-NMR and MRI Analysis

**Figure 1:**
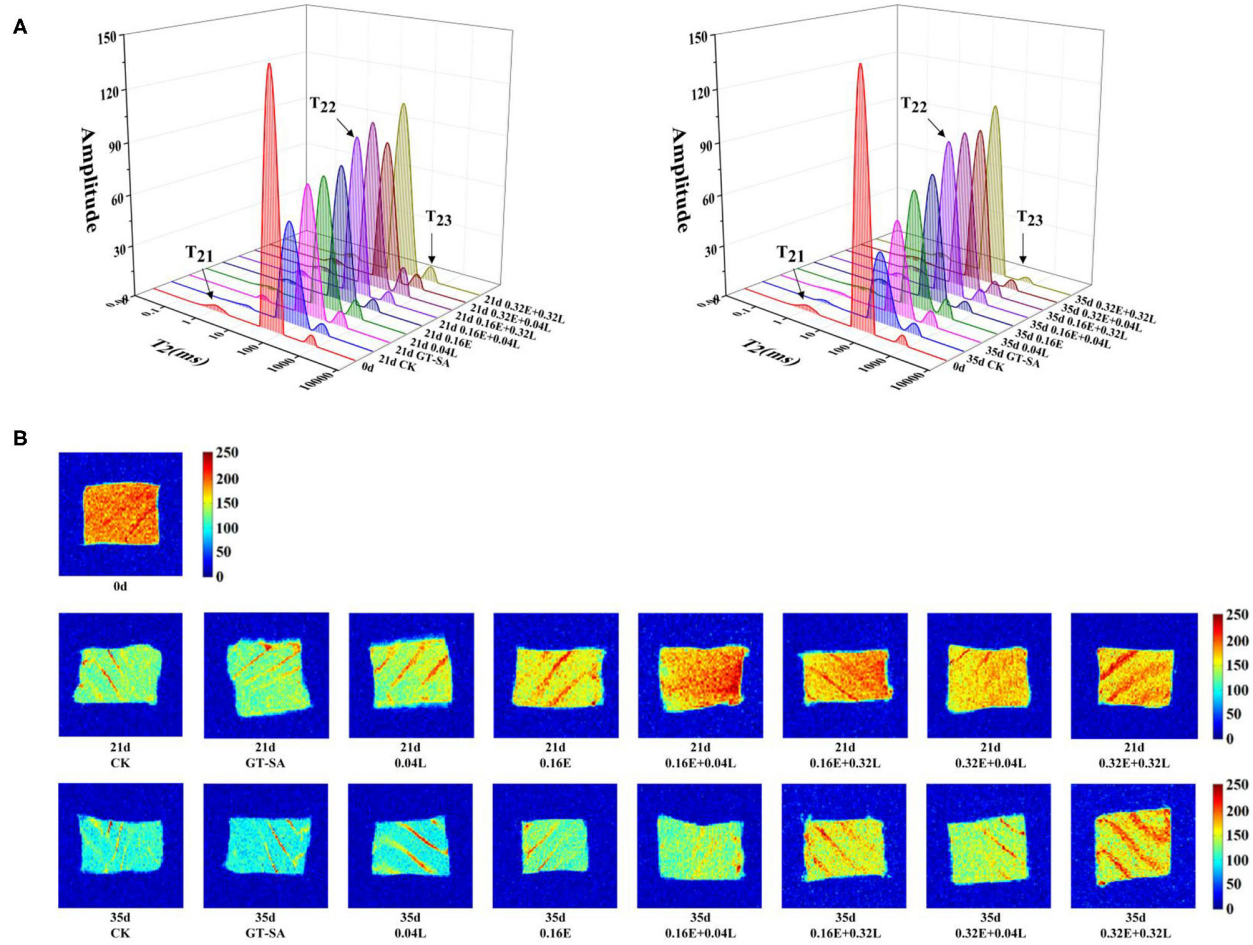
Changes in water distribution **(A)** and magnetic resonance imaging **(B)** of large yellow croaker samples during superchilling storage (CK, uncoated; GT-SA, GT-SA coating without preservatives; 0.04L, GT-SA coating with 0.04 mg·ml^−1^ LYS; 0.16E, GT-SA coating with 0.16 mg·ml^−1^ EGCG; 0.16E + 0.04L, GT-SA coating with 0.16 mg·ml^−1^ EGCG and 0.04 mg·ml^−1^ LYS; 0.16E + 0.32L, GT-SA coating with 0.16 mg·ml^−1^ EGCG and 0.32 mg·ml^−1^ LYS; 0.32E + 0.04L, GT-SA coating with 0.32 mg·ml^−1^ EGCG and 0.04 mg·ml^−1^ LYS; and 0.32E + 0.32L, GT-SA coating with 0.32 mg·ml^−1^ EGCG and 0.32 mg·ml^−1^ LYS).

Low-field nuclear magnetic resonance (LF-NM)R is a method to show the distribution of water in fish muscle and, thus, effectively evaluates their freshness (46). As shown in **Figure 3A**, three different types of peaks were distributed on the T_2_ relaxation time of large yellow croaker samples, corresponding to three different types of water: T_21_ (<10 ms) was defined as the bound water tightly associated with macromolecular hydrophilic groups, T_22_ (20–200 ms) was defined as the water fixed by physical forces in the structure of myogenic fibers, and T_23_ (200–1,000 ms) was defined as free water outside the myogenic fiber. In the present study, the transverse T_2_ reflected the binding force of samples of muscle to water, and the peak area indicated the content of different types of water (47). Compared with 0d, T_21_ and T_22_ decreased while T_23_ increased for all samples (Figure 1A). The fixed water content of the CK samples was lower than the other samples at any of sampling points. Some research also suggested that the fixed water could be released or converted to free water as the myofibrils were destroyed during the low temperature storage (48, 49). Active coatings containing EGCG or/and LYS could delay the rate of change of T_22_ and, thus, hinder the migration of fixed water, especially in the EGCG-LYS treated samples.

Magnetic resonance imaging (MRI) could provide some complementary visual information to study the water distribution and the migration of fish muscle (50). The corresponding pseudo-color images in large yellow croaker samples during superchilling storage are shown in Figure 1B, with the pseudo-color image appearing in red when the proton density is high, and in blue when the proton density is low. At any of the sampling points, the color of CK showed a less vibrant red than the other samples, demonstrating that the loss of water during superchilling storage was more severe in CK. Furthermore, the blue color of the EGCG-LYS treated samples were lower than the GT-SA, 0.04L, and 0.16E treated samples, which indicated that the active coatings containing EGCG and LYS were better able to maintain the quality for large yellow croaker samples during superchilling storage. The changes in MRI were consistent with LF-NMR results.

### Results of WHC

**Figure 2:**
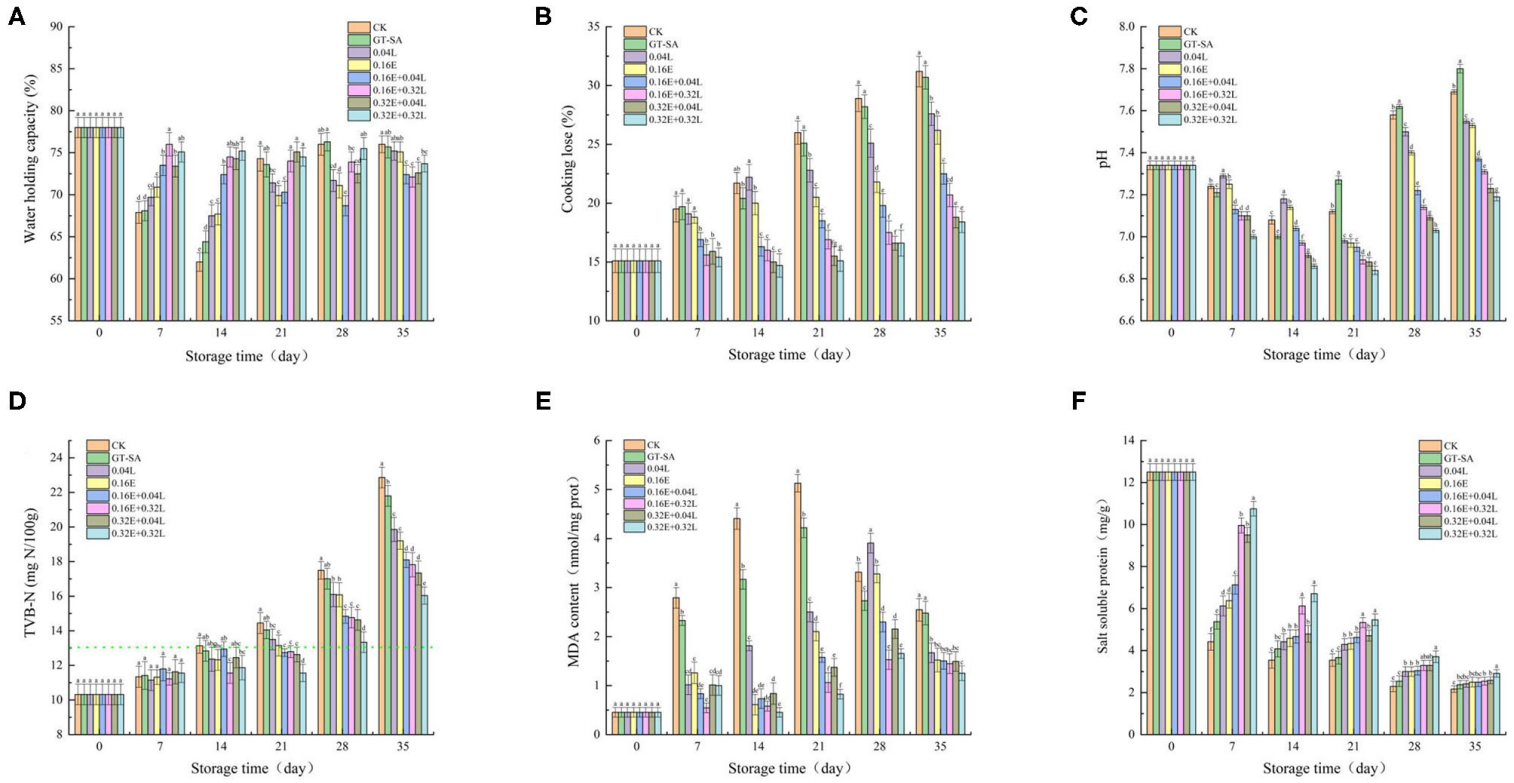
Changes in water holding capacity [WHC, **(A)**], cooking lose **(B)**, pH **(C)**, total volatile basic nitrogen values [TVB-N, **(D)**], malondialdehyde content [MDA, **(E)**], and salt soluble protein content **(F)** of large yellow croaker samples during superchilling storage (CK, uncoated; GT-SA, GT-SA coating without preservatives; 0.04L, GT-SA coating with 0.04 mg·ml^−1^ LYS; 0.16E, GT-SA coating with 0.16 mg·ml^−1^ EGCG; 0.16E + 0.04L, GT-SA coating with 0.16 mg·ml^−1^ EGCG, and 0.04 mg·ml^−1^ LYS; 0.16E + 0.32L, GT-SA coating with 0.16 mg·ml^−1^ EGCG and 0.32 mg·ml^−1^ LYS; 0.32E+0.04L: GT-SA coating with 0.32 mg·ml^−1^ EGCG and 0.04 mg·ml^−1^ LYS; and 0.32E + 0.32L, GT-SA coating with 0.32 mg·ml^−1^ EGCG and 0.32 mg·ml^−1^ LYS).

Water holding capacity (WHC) is an important quality indicator that reflects the level of degradation and of destruction of myofibril during low temperature storage (51). As shown in Figure 2A, the WHC values in 0.16E + 0.04L, 0.16E + 0.32L, 0.32E + 0.04L, and 0.32E + 0.32L samples were significantly higher than the other samples before 14 d of the superchilling storage. The decrease in WHC values reflected the drop in the water-locking capacity of the protein owing to the growth of spoilage bacterial and the action of endogenous autolytic enzymes during superchilling storage (52). However, after 14th day, the WHC of samples treated with CK, GT-SA, 0.04L, and 0.16E increased by different degrees, whereas the WHC of the EGCG-LYS treated samples were still decreasing. The fixed water of the non-compound preservative-treated samples could be released or converted into a free water in the middle and the late stages of superchilling storage, which will, therefore, result in less water separated from the experimental samples during centrifugation and with an unusual increase in WHC values. The results showed that the active coatings containing EGCG and LYS could enhance the water-holding capacity of large yellow croaker during superchilling storage, which were consistent with LF-NMR.

### Results of Cooking Loss

Cooking loss is remarkably an important parameter to evaluate the quality of superchilling food, as the loss of components during cooking affects the weight and the sensory quality of cooked fish (53). Some liquids and soluble substances are lost in the cooked fish, mainly due to the muscle structure damage caused by thermal denaturation of myofibrillar protein (54). The cooking loss of large yellow croaker on day 0 was 15.1% and showed an increased tendency during superchilling storage (Figure 2B). It could be seen that the cooking loss in 0.16E + 0.04L, 0.16E + 0.32L, 0.32E + 0.04L, and 0.32E + 0.32L samples were significantly lower than the other samples at any of the sampling points. The cooking loss for CK, GT-SA, 0.04L, 0.16E, 0.16E + 0.04L, 0.16E + 0.32L, 0.32E + 0.04L, and 0.32E + 0.32L were 31.2, 30.7, 27.6, 26.2, 22.5, 20.7, 18.8, and 18.4% on 35th day, respectively. Our results were in agreement with findings obtained by Chu et al. (55) for samples of large yellow croaker. It could be seen that the addition of GT-SA led to a relative reduction in the cooking loss values compared to CK. The formation of polysaccharide-protein complex through interactions between the GT-SA and the muscle protein inside large yellow croaker can be a reasonable reason for the decreasing cooking loss (56). Differences in the size of the ice crystals and the degree of muscle destruction in the different treated samples could make a difference to the cooking loss (30). With the combination of EGCG and LYS, the improvement of WHC and water distribution also leads to a decrease in the cooking loss. The results of cooking loss demonstrated that the EGCG-LYS treated samples had a minimal muscle structural damage in large yellow croaker superchilling storage, especially for the 0.32E + 0.32L samples.

### pH Analysis

Microbial activity is closely related to changes in pH values (57). The pH value of large yellow croaker samples on day 0 was 7.34, followed by a tendency to decrease and then increase (Figure 2C). The pH values of the EGCG–LYS treated samples were significantly lower than the other large yellow croaker samples. On 35th day, the pH values of the EGCG-LYS treated samples were at ~7.19–7.37, whereas the CK, GT-SA, 0.04L, and 0.16E samples increased to pH 7.53–7.80. The decrease in initial pH values was due to the release of lactic acid and inorganic phosphates during the process of glycolysis and ATP degradation (58). Nevertheless, the increase in pH values was accounted to the accumulation of volatile essential components, such as ammonia and amines that were produced by bacterial propagation (59). For the EGCG-LYS treated samples, the degradation of amino acids produced by spoilage bacterial propagation was significantly inhibited, leading to a decrease in biogenic amines production (58). Tan et al. (60) reported that pH change was an important factor for the freezing-induced myofibrillar protein denaturation, which could be reduced by attenuating the pH shift. In this study, the pH values of the EGCG-LYS treated samples were more stable than the other samples, indicating that the active coatings containing the compound preservatives might be able to reduce the myofibrillar protein denaturation and, thus, can better maintain the quality of large yellow croaker during superchilling storage. This is coherent with the results of microbiological indicators.

### TVB-N Analysis

**Figure 3:**
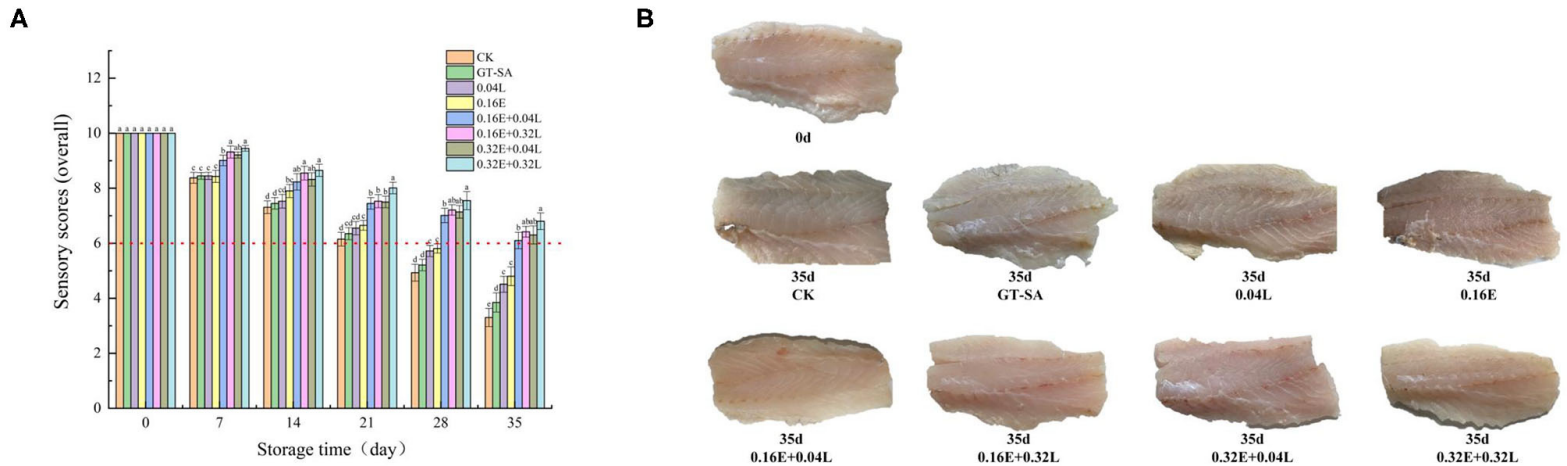
Changes in sensory score **(A)** and surface imaging **(B)** of large yellow croaker samples during superchilling storage (CK, uncoated; GT-SA, GT-SA coating without preservatives; 0.04L, GT-SA coating with 0.04 mg·mL^−1^ LYS; 0.16E, GT-SA coating with 0.16 mg·ml^−1^ EGCG; 0.16E + 0.04L, GT-SA coating with 0.16 mg·ml^−1^ EGCG and 0.04 mg·ml^−1^ LYS; 0.16E + 0.32L, GT-SA coating with 0.16 mg·ml^−1^ EGCG and 0.32 mg·ml^−1^ LYS; 0.32E + 0.04L, GT-SA coating with 0.32 mg·ml^−1^ EGCG and 0.04 mg·ml^−1^ LYS; and 0.32E + 0.32L, GT-SA coating with 0.32 mg·ml^−1^ EGCG and 0.32 mg·ml^−1^ LYS).

Total volatile basic nitrogen (TVB-N) is also indirectly related to microbial activity due to the basic compounds produced by microbial metabolism (61). The changes in TVB-N values of large yellow croaker samples are shown in Figure 2D. The TVB-N value of fresh large yellow croaker samples was 10.32 mg N/100 g, with a generally increasing tendency for all the samples during superchilling storage. Nevertheless, the TVB-N values increased significantly faster in the middle and late storage as a result of the increased endogenous enzymes activity and bacteria propagation (62). The usage of active coatings, containing compound preservatives, showed a significantly inhibitory effect on the increase of TVB-N values in large yellow croaker samples. As observed in this study, the TVB-N values for CK exceeded the first-grade freshness (13 mg/100 g) on 14th day (63), whereas the EGCG-LYS treated samples and the single preservative treated samples reached this level on days 28 and 21 of the storage period. In this research, the TVB-N values for CK, GT-SA, 0.04L, 0.16E, 0.16E + 0.04L, 0.16E + 0.32L, 0.32E + 0.04L, and 0.32E + 0.32L samples were 22.86, 21.80, 19.86, 19.2, 18.1, 17.82, 17.34, and 16.04 mg/100 g on the 35th day (Figure 3D), respectively. Since EGCG-LYS has a good inhibitory effect on the growth of spoilage bacteria (especially for Psychrophilic bacterial and *Pseudomonas* spp.), the active coating containing EGCG and LYS could alleviate the formation of TVB-N. This is similar to the results of Li et al. (24) who found that the combined LYS and resveratrol treated samples of coatings could restrain the increases in TVB-N values and maintain the quality of *Sciaenops ocellatus* during the chilled storage.

### MDA Analysis

Malondialdehyde (MDA) is a parameter for evaluating the degree of lipid oxidation (35). Lipid peroxides are produced by the reaction of oxygen free radicals and by the unsaturated fatty acids of fish, and are gradually decomposed into variety complex compounds, including MDA. The initial content of MDA in large yellow croaker samples was 0.46 nmol/mg prot, indicating high freshness and showed upward tendencies of all samples during the early and mid-late stage (Figure 2E). The MDA content in large yellow croaker generally increased to 5.13, 4.22, 2.50, 2.10, 1.58, 1.06, 1.37, and 0.82 nmol/mg prot in CK, GT-SA, 0.04L, 0.16E, 0.16E + 0.04L, 0.16E + 0.32L, 0.32E + 0.04L, and 0.32E + 0.32L samples on the 21st day, respectively, demonstrating that the active coatings containing EGCG and LYS could better delay lipid oxidation of large yellow croaker samples. The increase in MDA content during storage may be related to the increased oxidation rate of unsaturated fatty acids by the partial dehydration of large yellow croaker muscle (64). Subsequently, the MDA content showed decreasing tendencies at the later stage of storage in all samples, resulting to the reaction of MDA with other compounds, such as amino acids, nucleic acids, nucleosides, and proteins of phospholipids (65). Compared with CK, GT-SA, 0.04L, and 0.16E samples, the content of MDA in the EGCG-LYS treated samples were consistently lower during the superchilling storage. Liu et al. (66) reported that the antioxidant activity of EGCG was reduced with the presence of lysozyme. Because the antioxidant activity of polyphenolic compounds was closely related to their hydroxyl groups, the interaction between LYS and EGCG shielded the hydroxyl groups, resulting in a weakened hydrogen supply capacity and a decrease in the antioxidant capacity. However, the opposite conclusion was obtained in this study. This may be due, firstly, to the weak binding of LYS and EGCG, which did not have intermolecular covalent bonds (67), and secondly, to the fact that LYS could help the biodegradable coating containing polyphenol components to achieve a sustained release (68), which may be a valid reason for the synergistic effect of EGCG-LYS on antioxidant. Therefore, EGCG combined LYS as an antioxidant in the GT-SA active coatings and could retard the oxidation of unsaturated fatty acids, as well as improve the quality of large yellow croaker.

### Salt Soluble Protein Analysis

The content of salt soluble protein is highly correlated with the functional properties of the protein, such as gel properties, rheological properties, and emulsification properties. These properties only show up at high solubility (66). The content of salt soluble protein is shown in Figure 2F. Active coatings, containing EGCG and LYS, have noticeably prevented the reduction of salt soluble protein content compared with the CK samples. The content of salt soluble protein continued to decrease during superchilling storage, while the content of the EGCG-LYS treated samples were higher than the other samples at any of sampling points. In the early stage of superchilling storage, the salt soluble protein content was significantly reduced and the salt soluble protein content for CK, GT-SA, 0.04L, 0.16E, 0.16E + 0.04L, 0.16E + 0.32L, 0.32E + 0.04L, and 0.32E + 0.32L samples were 4.42, 5.38, 6.12, 6.38, 7.13, 9.96, 9.50, and 10.75 mg/g on the 7th day (Figure 2F), respectively. However, with the extension of the storage period, the difference in salt soluble protein content between different treated samples narrowed. Duun and Rustad (69) reported that the salt soluble protein content of superchilling cod filets was significantly reduced and was lower than that of ice storage samples at the same sampling points. In this study, it could be found that the salt soluble protein content was higher in the samples containing the active coating compared to CK. The decrease in salt soluble protein content implies the degradation of myofibrillar protein, which is related to protein oxidation and microbial activity. Since active coating is a barrier to the gas, moisture, and solute, the presence of a layer on the surface of the product reduces moisture and oxygen uptake to the large yellow croaker. Therefore, oxidation rate of myofibrillar protein could consequently be decreased (70). In addition, polysaccharides can enhance the bioavailability of EGCG by binding to protein-polyphenol complexes or by blocking the interaction between polyphenols and proteins, which will inhibit precipitation (16, 71). An increase in antimicrobial and antioxidant activities of EGCG-LYS treatments may lead to a better inhibition of myofibrillar protein degradation, which is a great benefit to improve the quality of the superchilling fish.

### FAAs and TAV Analysis

Free amino acids (FAAs) are important for the enhancement of flavors in fish products, including umami, bitterness, and sweetness. These also act as precursors to the production of harmful biogenic amines (for example, histamine can be produced by histidine in microbial metabolism) (72). Table 3 showed the content of FAAs in large yellow croaker filets on 0 and 35th day of superchilling storage. Most of the FAAs revealed increasing tendencies in all large yellow croaker samples during superchilling storage at −3°C. The main FAAs in large yellow croaker samples were glutamic acid, alanine, and lysine, accounting for 40.06–46.46% of the total AAs content, which contribute to the good flavor of large yellow croaker (74). Histidine was closely related to the formation of off-flavor in fish products, and in this study, its content accounted for 2.29–3.70% of total AAs content in all large yellow croaker samples. On day 0, the histidine content was 3.12 mg/100 ml in fresh samples and increased to 3.72–4.20 mg/100 ml in CK, GT-SA, 0.04L, and 0.16E samples on the 35th day. By contrast, the corresponding content in 0.16E + 0.04L, 0.16E + 0.32L, 0.32E + 0.04L, and 0.32E + 0.32L was 3.64, 3.26, 3.58, and 2.70 mg/100 mL, respectively, which is probably due to the great inhibition of spoilage bacteria growth by the EGCG-LYS treatments. Aspartic acid, Glutamic acid, glycine, and alanine are the sources of the characteristic flavor in aquatic products (75). The aspartic acid content in samples treated with CK, GT-SA, 0.04L, and 0.16E increased from 0.89 mg/100 ml on 0 day to 1.75–2.54 mg/100 ml on 35th day. The aspartic acid contents in the EGCG-LYS treated large yellow croaker samples had similar behaviors to the other treated samples, but their final content was significantly lower than others. Compared with the CK, the EGCG-LYS treated samples also showed a significant reduction in the glycine content during superchilling storage, similar to the results of Zhou et al. (76). The total FAAs content has increased significantly from 84.33 to 117.62–154.45 mg/100 ml. In addition, the sweet AAs showed the greatest increase compared to the changes in the umami, bitter, and tasteless AAs. Among these AAs, the sweet AAs and bitter AAs were the main flavor contributors, accounting for around 71.55% of the total AAs content. Indeed, FAAs are involved in a complex system of multiple metabolic pathways in fish, where it is consumed and produced at the same time as a key metabolite (77). Therefore, it is reasonable that different FAAs appear to the different fluctuations.

**Table 3:** Changes in free amino acids (FAAs) of large yellow croaker samples during superchilling storage.

**FAAs**	**Concentrations in large yellow croaker (mg/100 mL)**								
	**0 d**	**35 d**							
	**–**	**CK**	**GT-SA**	**0.04L**	**0.16E**	**0.16E + 0.04L**	**0.16E + 0.32L**	**0.32E + 0.04L**	**0.32E + 0.32L**
Aspartic acid	0.89 ± 0.01^f^	1.80 ± 0.06^c^	1.75 ± 0.12^c^	2.54 ± 0.09^a^	2.35 ± 0.08^b^	1.27 ± 0.22^d^	1.03 ± 0.02^ef^	1.14 ± 0.02^de^	1.05 ± 0.13^ef^
Threonine	3.18 ± 0.03^g^	5.75 ± 0.18^c^	6.13 ± 0.44^c^	7.77 ± 0.30^a^	6.92 ± 0.17^b^	4.47 ± 0.29^d^	3.97 ± 0.11^ef^	4.23 ± 0.01^de^	3.64 ± 0.16^f^
Serine	4.08 ± 0.06^c^	5.50 ± 0.20^abc^	4.46 ± 1.60^abc^	5.98 ± 0.32^a^	5.63 ± 0.04^ab^	4.64 ± 1.70^abc^	5.51 ± 0.21^abc^	5.41 ± 0.14^abc^	4.11 ± 0.06^bc^
Glutamic acid	9.84 ± 0.07^c^	13.16 ± 0.20^b^	13.43 ± 2.00^b^	16.83 ± 0.36^a^	11.95 ± 0.35^b^	9.78 ± 1.40^c^	12.08 ± 0.03^b^	16.30 ± 0.20^a^	12.15 ± 0.35^b^
Glycine	6.54 ± 0.01^f^	9.88 ± 0.03^a^	7.58 ± 0.60^de^	7.38 ± 0.24^de^	9.32 ± 0.19^ab^	8.34 ± 0.77^c^	7.83 ± 0.02^cd^	9.04 ± 0.11^b^	6.96 ± 0.14^ef^
Alanine	13.10 ± 0.06^d^	27.57 ± 0.23^a^	22.28 ± 1.90^c^	24.77 ± 0.22^b^	27.10 ± 0.50^a^	23.13 ± 0.89^c^	22.00 ± 0.06^c^	28.42 ± 0.53^a^	23.17 ± 0.42^c^
Valine	7.27 ± 0.04^c^	4.21 ± 3.00^d^	11.05 ± 0.40^b^	13.84 ± 0.08^a^	11.32 ± 0.57^b^	11.06 ± 1.89^b^	12.31 ± 0.01^ab^	12.99 ± 0.31^ab^	11.07 ± 0.48^b^
Methionine	3.97 ± 0.01^d^	5.74 ± 1.69^bcd^	16.16 ± 0.14^a^	6.58 ± 0.24^b^	6.08 ± 0.42^bc^	5.89 ± 2.62^bc^	6.42 ± 0.05^bc^	5.84 ± 0.03^bcd^	4.46 ± 0.47^cd^
Isoleucine	5.13 ± 0.04^c^	7.22 ± 2.10^bc^	7.67 ± 0.62^bc^	10.36 ± 0.21^a^	7.95 ± 0.03^ab^	7.69 ± 3.41^bc^	8.37 ± 0.04^ab^	9.09 ± 0.19^ab^	7.26 ± 0.13^bc^
Leucine	8.17 ± 0.02^d^	11.44 ± 3.45^c^	12.12 ± 1.12^c^	16.22 ± 0.26^a^	12.81 ± 0.02^c^	12.57 ± 1.07^bc^	13.17 ± 0.06^bc^	14.72 ± 0.22^ab^	11.63 ± 0.25^bc^
Tyrosine	3.21 ± 0.07^c^	4.63 ± 0.53^bc^	5.02 ± 0.42^ab^	6.45 ± 0.05^a^	5.28 ± 0.08^ab^	5.15 ± 2.52^ab^	5.13 ± 0.12^ab^	5.55 ± 0.18^ab^	4.26 ± 0.13^bc^
Phenylalanine	2.58 ± 0.01^b^	10.40 ± 3.25^ab^	4.22 ± 0.32^ab^	5.55 ± 0.13^a^	4.24 ± 0.16^ab^	4.20 ± 2.28^ab^	4.14 ± 0.03^ab^	4.34 ± 0.17^ab^	3.28 ± 0.07^ab^
Lysine	13.00 ± 0.08^f^	20.51 ± 2.81^ab^	19.56 ± 1.40^bc^	21.77 ± 0.06^a^	17.16 ± 0.20^de^	16.50 ± 0.10^de^	15.99 ± 0.05^e^	18.18 ± 0.35^cd^	19.32 ± 0.37^bc^
Histidine	3.12 ± 0.04^cd^	4.20 ± 0.82^a^	3.72 ± 0.23^bc^	3.92 ± 0.01^ab^	3.96 ± 0.11^ab^	3.64 ± 0.04^bc^	3.26 ± 0.05^c^	3.58 ± 0.07^bc^	2.70 ± 0.07^d^
Proline	0.42 ± 0.33^b^	2.31 ± 1.41^ab^	3.00 ± 1.34^a^	4.48 ± 0.67^a^	2.88 ± 0.80^a^	3.07 ± 2.34^a^	2.53 ± 1.06^ab^	3.18 ± 0.65^a^	2.56 ± 0.35^ab^
Total AA	84.33 ± 0.63^g^	133.98 ± 0.02^d^	137.95 ± 3.45^c^	154.45 ± 1.81^a^	134.95 ± 0.01^d^	121.41 ± 0.92^e^	123.73 ± 0.11^e^	142.00 ± 1.07^b^	117.62 ± 2.07^f^
Umani AA	10.73 ± 0.08^c^	14.96 ± 0.26^c^	15.18 ± 2.12^c^	19.37 ± 0.45^a^	14.29 ± 0.43^cd^	11.04 ± 1.62^c^	13.11 ± 0.05^d^	17.44 ± 0.22^b^	13.20 ± 0.48^d^
Sweet AA	26.90 ± 0.16^c^	48.71 ± 0.64^a^	40.45 ± 4.54^b^	45.90 ± 1.08^a^	48.97 ± 0.90^a^	40.59 ± 3.65^b^	39.30 ± 0.40^b^	47.10 ± 0.79^a^	37.88 ± 0.78^b^
Bitter AA	33.44 ± 0.23^d^	47.49 ± 14.84^bc^	59.76 ± 3.25^ab^	62.91 ± 0.98^a^	51.65 ± 1.39^abc^	50.20 ± 13.83^abc^	52.80 ± 0.36^abc^	56.10 ± 1.17^abc^	44.66 ± 1.60^cd^
Tasteless AA	13.25 ± 0.41^d^	22.82 ± 4.22^b^	22.56 ± 2.74^b^	26.26 ± 0.73^a^	20.04 ± 1.00^bc^	19.57 ± 2.44^bc^	18.52 ± 1.11^c^	21.35 ± 1.00^bc^	21.88 ± 0.72^bc^

*Total AAs, total of all FAAs; Umami AAs, aspartic acid + glutamic acid; Sweet AAs, threonine + serine + alanine + glycine; Bitter AAs, valine + methionine + isoleucine + leucine + phenylalanine + tyrosine + histidine; Tasteless AAs, lysine + proline (73). CK, uncoated; GT-SA, GT-SA coating without preservatives; 0.04L, GT-SA coating with 0.04 mg·ml^−1^ LYS; 0.16E, GT-SA coating with 0.16 mg·ml^−1^ EGCG; 0.16E + 0.04L, GT-SA coating with 0.16 mg·ml^−1^ EGCG and 0.04 mg·ml^−1^ LYS; 0.16E + 0.32L, GT-SA coating with 0.16 mg·ml^−1^ EGCG and 0.32 mg·ml^−1^ LYS; 0.32E + 0.04L, GT-SA coating with 0.32 mg·ml^−1^ EGCG and 0.04 mg·ml^−1^ LYS; and 0.32E + 0.32L, GT-SA coating with 0.32 mg·ml^−1^ EGCG and 0.32 mg·ml^−1^ LYS*.

*Same superscript letters for mean values within a column indicate no significant differences (P>0.05)*.

The ratio of certain flavor compound content to its threshold value is known as the TAV (78). The contribution of FAAs in each food matrix is determined by their TAVs (79). Table 4 unfolded the TAVs in large yellow croaker samples on the 0 and 35th day of superchilling storage. According to the TAVs, glutamic acid and alanine were the predominant amino acids flavor in large yellow croaker samples, accounting for around 28.39% of the total AAs content, followed by lysine, methionine, and valine even though all of the TAVs were <1. The TAVs of FAAs of samples treated with different treatments showed different trends. Generally, the GT-SA active coatings containing EGCG and LYS could accumulate the TAVs of some umami AAs and could reduce the TAVs of bitter AAs, which enhanced the flavor quality of large yellow croaker samples during superchilling storage.

**Table 4:** Changes in taste activity values (TAVs) of large yellow croaker samples during superchilling storage.

**FAAs**	**Taste threshold (mg/100 mL)**	**TAVs in large yellow croaker**								
		**0 d**	**35 d**							
		**–**	**CK**	**GT-SA**	**0.04L**	**0.16E**	**0.16E + 0.04L**	**0.16E + 0.32L**	**0.32E + 0.04L**	**0.32E + 0.32L**
Aspartic acid	100	<0.10	<0.10	<0.10	<0.10	<0.10	<0.10	<0.10	<0.10	<0.10
Threonine	260	<0.10	<0.10	<0.10	<0.10	<0.10	<0.10	<0.10	<0.10	<0.10
Serine	150	<0.10	<0.10	<0.10	<0.10	<0.10	<0.10	<0.10	<0.10	<0.10
Glutamic acid	30	0.33 ± 0.00^c^	0.44 ± 0.01^b^	0.45 ± 0.07^b^	0.56 ± 0.01^a^	0.40 ± 0.01^b^	0.33 ± 0.05^c^	0.40 ± 0.00^b^	0.54 ± 0.01^a^	0.41 ± 0.01^b^
Glycine	130	<0.10	<0.10	<0.10	<0.10	<0.10	<0.10	<0.10	<0.10	<0.10
Alanine	60	0.22 ± 0.00^d^	0.46 ± 0.00^a^	0.37 ± 0.03^c^	0.41 ± 0.00^b^	0.45 ± 0.01^a^	0.39 ± 0.01^bc^	0.37 ± 0.00^c^	0.47 ± 0.01^a^	0.39 ± 0.01^bc^
Valine	40	0.18 ± 0.00^c^	0.26 ± 0.08^b^	0.28 ± 0.01^b^	0.35 ± 0.00^a^	0.28 ± 0.01^b^	0.28 ± 0.05^b^	0.31 ± 0.00^ab^	0.32 ± 0.01^ab^	0.28 ± 0.01^b^
Methionine	30	0.13 ± 0.00^c^	0.19 ± 0.06^bc^	0.54 ± 0.00^a^	0.22 ± 0.01^b^	0.20 ± 0.01^bc^	0.20 ± 0.09^bc^	0.21 ± 0.00^b^	0.19 ± 0.00^bc^	0.15 ± 0.02^bc^
Isoleucine	90	<0.10	<0.10	<0.10	0.12 ± 0.00^a^	<0.10	<0.10	<0.10	0.10 ± 0.00^b^	<0.10
Leucine	190	<0.10	<0.10	<0.10	<0.10	<0.10	<0.10	<0.10	<0.10	<0.10
Tyrosine	N.T	N.T	N.T	N.T	N.T	N.T	N.T	N.T	N.T	N.T
Phenylalanine	90	<0.10	<0.10	<0.10	<0.10	<0.10	<0.10	<0.10	<0.10	<0.10
Lysine	50	0.26 ± 0.00^e^	0.41 ± 0.06^ab^	0.39 ± 0.03^bc^	0.44 ± 0.00^a^	0.34 ± 0.00^d^	0.33 ± 0.00^d^	0.32 ± 0.00^d^	0.36 ± 0.01^cd^	0.39 ± 0.01^bcd^
Histidine	20	0.16 ± 0.00^c^	0.21 ± 0.04^a^	0.18 ± 0.01^bc^	0.20 ± 0.00^ab^	0.20 ± 0.00^ab^	0.18 ± 0.01^bc^	0.16 ± 0.00^c^	0.18 ± 0.00^bc^	0.13 ± 0.00^d^
Proline	300	<0.10	<0.10	<0.10	<0.10	<0.10	<0.10	<0.10	<0.10	<0.10

*N.T, no taste threshold parameters were found in previous research. Taste threshold values of free amino acids were according to Qi et al. (36). CK, uncoated; GT-SA, GT-SA coating without preservatives; 0.04L, GT-SA coating with 0.04 mg·ml^−1^ LYS; 0.16E, GT-SA coating with 0.16 mg·ml^−1^ EGCG; 0.16E + 0.04L, GT-SA coating with 0.16 mg·ml^−1^ EGCG and 0.04 mg·ml^−1^ LYS; 0.16E + 0.32L, GT-SA coating with 0.16 mg·ml^−1^ EGCG and 0.32 mg·ml^−1^ LYS; 0.32E + 0.04L, GT-SA coating with 0.32 mg·ml^−1^ EGCG and 0.04 mg·ml^−1^ LYS; and 0.32E + 0.32L, GT-SA coating with 0.32 mg·ml^−1^ EGCG and 0.32 mg·ml^−1^ LYS*.

*Same superscript letters for mean values within a column indicate no significant differences (P>0.05)*.

### VOCs

In large yellow croaker superchilling storage, a total of twenty VOCs, including alcohols, ketones, esters, acids, and phthalan, were discovered and measured, disregarding amines, hydrocarbons, and aromatic compounds (Table 5) (80). The majority of VOCs exhibited increased tendencies during superchilling storage, while others showed decreased tendencies. Furthermore, the partial disappearance of VOCs of fresh large yellow croaker samples at the end of superchilling storage could be observed in the present and in other researches (48). The peak areas of 3-methyl-1-butanol, 3-(methylthio)-1-propanol, 2-octanol, non-anal, 2-octanone, and 3-methyl-pentanoic acid were larger than the other VOCs.

**Table 5:** Retention index and area of main volatile compounds identified in large yellow croaker after 0 and 35 days of superchilling storage.

**Name**	**RI**	**VOCs in large yellow croaker**								
		**0 d**	**35 d**							
		**–**	**CK**	**GT-SA**	**0.04L**	**0.16E**	**0.16E + 0.04L**	**0.16E + 0.32L**	**0.32E + 0.04L**	**0.32E + 0.32L**
**Alcohols**										
1-Butanol, 3-methyl-	697	1.39 × 10^8^	2.45 × 10^7^	1.97 × 10^8^	1.40 × 10^8^	1.85 × 10^8^	1.95 × 10^8^	8.36 × 10^7^	1.32 × 10^8^	1.47 × 10^8^
1-Heptanol	960	N.D	1.55 × 10^6^	N.D	9.91 × 10^5^	N.D	N.D	N.D	N.D	N.D
1-Octanol	1,059	5.56 × 10^5^	2.42 × 10^6^	N.D	N.D	N.D	N.D	N.D	3.22 × 10^5^	3.50 × 10^5^
1-Octen-3-ol	969	N.D	3.00 × 10^6^	N.D	N.D	N.D	3.77 × 10^5^	N.D	N.D	N.D
1-Propanol, 3-(methylthio)-	912	3.44 × 10^6^	5.29 × 10^6^	3.82 × 10^6^	3.63 × 10^6^	3.86 × 10^6^	8.30 × 10^6^	1.75 × 10^6^	2.79 × 10^6^	3.41 × 10^6^
2-Hexanol, 5-methyl-	879	N.D	N.D	1.48 × 10^6^	N.D	N.D	N.D	N.D	7.30 × 10^5^	N.D
2-Octanol	979	7.09 × 10^6^	1.73 × 10^7^	6.12 × 10^6^	3.04 × 10^6^	8.49 × 10^6^	8.75 × 10^6^	8.82 × 10^6^	8.87 × 10^6^	5.05 × 10^6^
2-Penten-1-ol, (Z)-	769	N.D	2.25 × 10^6^	N.D	2.13 × 10^6^	N.D	7.08 × 10^5^	N.D	N.D	N.D
4,5-Octanediol, 2,7-dimethyl-	1,212	N.D	8.64 × 10^6^	N.D	N.D	5.70 × 10^6^	3.77 × 10^6^	N.D	N.D	N.D
**Ketones**										
2-Heptanone	853	N.D	6.00 × 10^6^	7.13 × 10^6^	6.91 × 10^5^	N.D	N.D	N.D	N.D	3.89 × 10^5^
2-Nonanone	1,052	N.D	N.D	N.D	1.70 × 10^6^	1.61 × 10^6^	N.D	N.D	N.D	N.D
2-Octanone	952	3.12 × 10^6^	2.38 × 10^6^	4.10 × 10^6^	N.D	N.D	5.71 × 10^6^	3.43 × 10^6^	2.98 × 10^6^	N.D
Acetoin	717	N.D	4.04 × 10^6^	N.D	1.92 × 10^5^	9.87 × 10^5^	1.11 × 10^6^	N.D	N.D	N.D
**Esters**										
Hexanethioic acid, S-methyl ester	1,104	N.D	N.D	3.49 × 10^6^	N.D	N.D	N.D	N.D	1.48 × 10^6^	N.D
Octanethioic acid, S-methyl ester	1,303	N.D	N.D	1.04 × 10^6^	N.D	N.D	N.D	N.D	2.71 × 10^5^	1.23 × 10^6^
Pentanoic acid, 4-methyl-2-oxo-,	956	5.95 × 10^5^	5.16 × 10^6^	8.34 × 10^5^	7.21 × 10^5^	4.78 × 10^6^	2.61 × 10^6^	9.15 × 10^5^	3.58 × 10^5^	N.D
methyl ester										
**Acids**										
Butanoic acid, 3-methyl-	811	N.D	N.D	2.29 × 10^7^	N.D	N.D	N.D	N.D	N.D	6.71 × 10^6^
Pentanoic acid, 3-methyl-	910	1.85 × 10^6^	5.38 × 10^6^	N.D	3.88 × 10^6^	1.01 × 10^7^	2.85 × 10^7^	7.04 × 10^6^	5.00 × 10^6^	N.D
Propanoic acid, 2-methyl-	711	5.15 × 10^5^	9.70 × 10^5^	N.D	4.73 × 10^5^	9.83 × 10^5^	2.93 × 10^6^	1.20 × 10^6^	8.70 × 10^5^	1.83 × 10^6^
**Others**										
Phthalan	1,036	N.D	1.03 × 10^5^	N.D	N.D	N.D	N.D	5.36 × 10^5^	N.D	9.14 × 10^5^

*N.D, not detected*.

A total of six alcohols were detected in large yellow croaker samples. Among these alcohols, the compounds of 3-methyl-1-butanol, 3-(methylthio)-1-propanol, and 2-octanol were found in all samples, while others were not present in all samples. Balsamic is an odor descriptor used to define the aroma of 3-methyl-1-butanol (probably originated from the process of oxidative deamination of leucine) in literature study (81). Generally, volatile saturated alcohols do not contribute much to food flavor due to their high thresholds, except when they are present at high levels (82). In contrast, as the low thresholds, the unsaturated alcohols could play an important role in the faint scent of fresh fish. The high contents of 1-octen-3-ol and (Z)-2-penten-1-ol were found in the detected large yellow croaker samples (Table 5). The 1-octen-3-ol is a significant off-flavor contributor and is considered to be an indicator of lipid oxidation (83). Furthermore, the (Z)-2-penten-1-ol has a grilled hazel nut odor and is a main volatile alcohol in turbot (84).

Four ketones were found in large yellow croaker samples (Table 5). Ketones can be produced through the degradation of amino acids and polyunsaturated fatty acids, and also through bacterial metabolism. In addition, ketones with a low aroma threshold contribute to fishy odors (82). Acetoin is thought to be a potential signature for salmon spoilage (85), and its formation in fish products has been linked to lactic acid bacteria (86), *Shewanella baltica* (87), and *Brochothrix thermosphacta* (88). Compared with the CK treated samples, the active coatings containing EGCG or/and LYS treated samples had significant lower peak areas of acetoin, especially for the EGCG-LYS treated samples.

Three esters and three acids were detected in the large yellow croaker filets (Table 5). Most of the esters contribute mainly to the fruity and sweet aroma (89). Sulfur-containing compounds are considered important odor-active components (79). In this study, sulfur-containing esters were only found in the active coatings treated samples. The VOCs produced during the lipid auto-oxidation process are widely regarded as one of the main causes of degradation in fish quality (90). As shown by the changes in VOCs, the EGCG-LYS treatments had an inhibitory effect on both lipid oxidation and on microbial growth in large yellow croaker due to the low peak area of off-flavor compounds.

### Sensory Assessment

Figure 3A shows the overall sensory score of large yellow croaker samples during superchilling storage at −3°C for 35 days. On 0 day, the fresh large yellow croaker samples obtained the highest sensory score, suggesting a good quality. However, with the increasing storage time, the sensory scores of all samples showed decreasing tendencies. The organoleptic results revealed that the sensory scores of the EGCG-LYS treated large yellow croaker samples were significantly higher than the other samples. On the 28th day, CK samples had poor color, odor, and texture with the sensory score below 6, which was considered as an unacceptable value for large yellow croaker samples in the current research, while only the EGCG-LYS treated samples still had high levels of quality. On the 35th day, the scores of 0.16E + 0.04L, 0.16E + 0.32L, 0.32E + 0.04L, and 0.32E + 0.32L samples were 6, 6.42, 6.30, and 6.80, respectively, indicating that the quality was still acceptable. The surface imaging of Figure 3B showed that CK, GT-SA, 0.04L, and 0.16E samples had obvious black change in color compared with 0.16E + 0.04L, 0.16E + 0.32L, 0.32E + 0.04L, and 0.32E + 0.32L samples on the 35th day of superchilling. Thus, using the GT-SA active coatings containing EGCG and LYS is an effective method to delay the deterioration and to improve the organoleptic quality for large yellow croaker samples, especially for the 0.32E + 0.32L treated samples. This was supported by the analysis of microbiological and physicochemical indicators.

## Conclusions

The synergistic interactions effect of 0.5% GT and 1.5% SA coatings, containing different natural preservatives (EGCG and LYS) on the quality enhancement in large yellow croaker samples in superchilling storage at −3°C, was investigated in this study. The microbiological, physicochemical, and flavor results demonstrated that the GT-SA active coatings containing EGCG and LYS prolong the shelf life for at least 7 days and improve the safety of large yellow croaker due to its antimicrobial and antioxidant activity. Furthermore, the 0.32E + 0.32L treated samples showed the best preservation effect in all groups during superchilling storage. This is primarily caused by the stronger preservation effect of EGCG and LYS at high concentrations. Therefore, GT-SA active coatings supplemented with EGCG and LYS treatments are appropriate ways to improve the quality of large yellow croaker samples during superchilling storage, where extended storage times may be required.

## Data Availability Statement

The raw data supporting the conclusions of this article will be made available by the authors, without undue reservation.

## Author Contributions

JP and JM: conceptualization, investigation, and writing-original draft. JP and HY: data curation and formal analysis. JX: funding acquisition and validation. JP, JM, WQ, and JX: methodology. JM and JX: project administration and writing-review and editing. JP and WQ: software. All authors contributed to the article and approved the submitted version.

## Funding

This research was funded by the China Agriculture Research System (CARS-47), Capacity Building project of Local Colleges of Shanghai Science and Technology Commission (21010502100), Shanghai Science and Technology Key Project on Agriculture from Shanghai Municipal Agricultural Commission (2019-02-08-00-10-F01143), National Key Research and Development Program (2016YFD0400106), and Shanghai Science and Technology Commission Platform Capacity Construction Project (19DZ2284000).

## Conflict of Interest

The authors declare that the research was conducted in the absence of any commercial or financial relationships that could be construed as a potential conflict of interest.

## Publisher's Note

All claims expressed in this article are solely those of the authors and do not necessarily represent those of their affiliated organizations, or those of the publisher, the editors and the reviewers. Any product that may be evaluated in this article, or claim that may be made by its manufacturer, is not guaranteed or endorsed by the publisher.
